# 130 Comparing Artificial Intelligence Guided Image Assessment to Current Methods of Burn Assessment

**DOI:** 10.1093/jbcr/irae036.129

**Published:** 2024-04-17

**Authors:** Justin J Lee, Mahla Abdolahnejad, Alexander Morzycki, Hannah O Chan, Rakesh Joshi, Collin Hong, Joshua N Wong

**Affiliations:** University of Alberta, Edmonton, Alberta; Skinopathy, Toronto, Ontario; University of Washington, Harborview Burn Centre, Edmonton, AB; University of Alberta Burn Centre, Edmonton, AB; University of Alberta, Edmonton, Alberta; Skinopathy, Toronto, Ontario; University of Washington, Harborview Burn Centre, Edmonton, AB; University of Alberta Burn Centre, Edmonton, AB; University of Alberta, Edmonton, Alberta; Skinopathy, Toronto, Ontario; University of Washington, Harborview Burn Centre, Edmonton, AB; University of Alberta Burn Centre, Edmonton, AB; University of Alberta, Edmonton, Alberta; Skinopathy, Toronto, Ontario; University of Washington, Harborview Burn Centre, Edmonton, AB; University of Alberta Burn Centre, Edmonton, AB; University of Alberta, Edmonton, Alberta; Skinopathy, Toronto, Ontario; University of Washington, Harborview Burn Centre, Edmonton, AB; University of Alberta Burn Centre, Edmonton, AB; University of Alberta, Edmonton, Alberta; Skinopathy, Toronto, Ontario; University of Washington, Harborview Burn Centre, Edmonton, AB; University of Alberta Burn Centre, Edmonton, AB; University of Alberta, Edmonton, Alberta; Skinopathy, Toronto, Ontario; University of Washington, Harborview Burn Centre, Edmonton, AB; University of Alberta Burn Centre, Edmonton, AB

## Abstract

**Introduction:**

Appropriate identification of burn depth and size is paramount. Despite the development of assessment aids [e.g., laser doppler imaging (LDI)], clinical assessment remains a gold standard, which assesses partial thickness burn depth with ~67% accuracy. We sought to develop an image-based artificial intelligence system that predicts burn severity and wound margins for use as a triaging tool in the community.

**Methods:**

Modified EfficientNet architecture trained by 1684 mobile-device-captured burn datasets of different burn depths were previously utilized to create a convoluted neural network (CNN). The CNN was modified to a novel Boundary-Attention Mapping (BAM) algorithm using elements of saliency mapping, which was utilized to recognize the boundaries of burns. For validation, 144 patient charts that included clinical assessment, burn location, total body surface area, and LDI assessment were retrieved for a retrospective study at an academic burn center. The clinical images underwent CNN-BAM assessment and were directly compared with the LDI assessment.

**Results:**

A CNN using a four-level burn severity classification achieved an accuracy of 85% (micro/macro-averaged ROC scores). The CNN-BAM system can successfully highlight burns from surrounding tissue with high confidence. Our method's burn area segmentations attained an accuracy of 91.60%, sensitivity of 78.17%, and specificity of 93.37%, when compared to LDI methodology and conducting a pixel-wise comparison of LDI’s from 104 patients (Figure 1). Results comparing the CNN-BAM outputs to clinical and LDI assessments have shown a high degree of correlation between the CNN-BAM burn severity predictions to those extrapolated from LDI healing potential (66% agreement Cohen’s Kappa analysis). This is in comparison to clinical vs LDI assessment, which had 28% agreement, and clinical vs CNN-BAM, which had 32% agreement (Figure 2).

**Conclusions:**

CNN-BAM algorithm gives equivalent accuracy in detecting burn depth as LDI with a more economical and accessible application when embedded in a mobile device.

**Applicability of Research to Practice:**

CNN-BAM algorithm can be embedded in a mobile application, which can be easily accessible to healthcare providers to rapidly assess burn depth. This can be especially useful in rural communities to accurately assess and transfer patients to specialized burn centres and reduce unnecessary transfers when injuries are less severe.

